# A novel haplotype of low-frequency variants in the aldosterone synthase gene among northern Han Chinese with essential hypertension

**DOI:** 10.1097/MD.0000000000008150

**Published:** 2017-09-29

**Authors:** Hao Zhang, Xueyan Li, Li Zhou, Keyong Zhang, Qi Zhang, Jingping Li, Ningning Wang, Ming Jin, Nan Wu, Mingyu Cong, Changchun Qiu

**Affiliations:** a Institute of Polygenic Disease, Qiqihar Medical University, Qiqihar, Heilongjiang Province; b Institute of Basic Medical Sciences, Chinese Academy of Medical Sciences (Peking Union) Medical College (CAMS/PUMC), Beijing, P. R. China.

**Keywords:** *CYP11B2*, essential hypertension, genetics, haplotype, low-frequency variants

## Abstract

Low-frequency variants showed that there is more power to detect risk variants than to detect protective variants in complex diseases. Aldosterone plays an important role in the renin–angiotensin–aldosterone system, and aldosterone synthase catalyzes the speed-controlled steps of aldosterone biosynthesis. Polymorphisms of the aldosterone synthase gene (*CYP11B2*) have been reported to be associated with essential hypertension (EH). CYP11B2 polymorphisms such as –344T/C, have been extensively reported, but others are less well known. This study aimed to assess the association between human CYP11B2 and EH using a haplotype-based case–control study. A total of 1024 EH patients and 956 normotensive controls, which consist of north Han population peasants, were enrolled. Seven single nucleotide polymorphisms (SNPs) (rs28659182, rs10087214, rs73715282, rs542092383, rs4543, rs28491316, and rs7463212) covering the entire human *CYP11B2* gene were genotyped as markers using the MassARRAY system. The major allele G frequency of rs542092383 was found to be risk against hypertension [odds ratio (OR) 3.478, 95% confidence interval (95% CI) 1.407–8.597, *P* = .004]. The AG genotype frequency of SNP rs542092383 was significantly associated with an increased risk of hypertension (OR 4.513, 95% CI 1.426–14.287, *P* = .010). In the haplotype-based case–control analysis, the frequency of the T-G-T haplotype was higher for EH patients than for controls (OR 5.729, 95% CI 1.889–17.371, *P* = .000495). All |D′| values of the seven SNPs were >0.9, and *r*^2^ values for rs28659182- rs10087214-rs28491316-rs7463212 SNPs were >0.8 and showed strong linkage intensity. Haplotype T-G-T may therefore be a useful genetic marker for EH.

## 1 Introduction

Single nucleotide polymorphisms (SNPs) discovered by genome-wide association studies (GWAS), are significantly associated with many complex traits, yet account for only a small fraction of the genetic variation of complex traits in human populations. Where is the missing heritability? There is a evidence that the remaining heritability is due to incomplete linkage disequilibrium (LD) between causal variants and genotyped SNPs, exacerbated by causal variants having lower minor allele frequency (MAF) than the SNPs explored to date.^[1]^

Most complex diseases involve in a mix of genetic and environmental factors, and much of the heritability remains unaccounted for by common variants (≥5% frequency). It has been postulated that lower frequency variants contribute to the remaining heritability. Low-frequency (<5% frequency) variants showed that there is more power to detect risk variants than to detect protective variants.^[2]^

Essential hypertension (EH) is a kind of complex diseases and a major risk factor for diseases such as cardiovascular disease and kidney failure.^[3–5]^ The renin–angiotensin–aldosterone system (RAAS) plays an important role in modulating blood pressure (BP) in EH by regulating sodium and intravascular volume homeostasis.^[6,7]^ Aldosterone is a key component of the RAAS.^[8,9]^ Aldosterone synthase, a member of the cytochrome P450 superfamily of enzymes, is encoded by *CYP11B2*, which is spread over 7 kb on chromosome 8q24.3, and contains 9 exons. It catalyzes the terminal steps of steroid biosynthesis, in which 11-deoxycorticosterone is converted to aldosterone.^[10,11]^ Therefore, human *CYP11B2* is a potential candidate gene for the development of EH.

Previous studies have shown that *CYP11B2* may be involved in EH,^[12,13]^ and a number of *CYP11B2* polymorphisms have been identified such as C-344T, K173R, and intron 2 IC. Among them, C-344T in the promoter region has been widely studied, but the results are often inconsistent.^[14,15]^ Studies on other *CYP11B2* polymorphisms are rare, so this study aimed to investigate the association between novel *CYP11B2* polymorphisms and susceptibility to EH in the Han population of northern China, and we found some low-frequency variants that may be a more powerful marker in EH.

## 2 Methods

### 2.1 Study population

All participants were recruited by way of health examination between July 2012 and July 2015. Informed consent was obtained from each participant and all protocols were previously approved by the Ethics Committee of Qiqihar University. All participants were of northern Han Chinese origin, within 3 generations, and currently reside in Lanxi County (46° 20′ 0″ N, 126° 16′ 0″ E) of Heilongjiang Province, in the north of China.

A total of 1024 unrelated patients (509 males and 515 females) with EH and 956 normotensive subjects (296 males and 660 females) were included in this investigation if they met the following inclusion criteria. Subjects who consistently had a systolic BP (SBP) ≥140 mm Hg and/or diastolic BP (DBP) ≥100 mm Hg or with a history of hypertension were diagnosed as hypertensives. The control group was composed of 956 subjects with SBP <120 mm Hg and DBP < 80 mm Hg, without a history of hypertension, and minor illness patients without hypertension, hyperlipidemia, diabetes mellitus, tumors, or a history of family hypertension in their previous records. Patients with secondary hypertension or pregnancy were excluded from this study.

BP was measured using a Riva–Rocci sphygmomanometer. Subjects rested for 10 minutes, and then their BP was measured 3 times, 5 minutes apart, on the right arm by experienced doctors according to standard protocols recommended by the American Heart Association.^[16]^ The 3 consecutive values were averaged to match inclusion criteria and analysis. Body mass index was calculated by measuring height and weight without shoes and wearing light clothing.

### 2.2 SNP selection and genotyping

**Figure 1:**
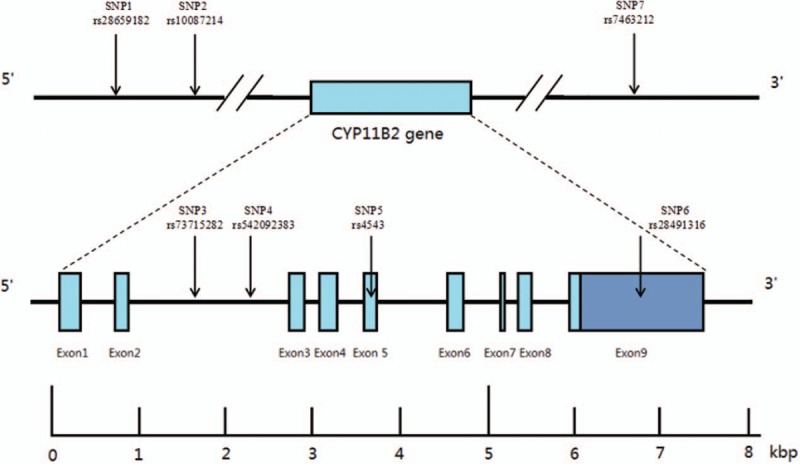
Structure of human *CYP11B2*. The gene consists of 9 exons (boxes) separated by 8 introns (lines). Arrows indicate the location of single nucleotide polymorphisms. kbp = kilobase pairs.

Selection of human *CYP11B2* gene SNP was based on previous results of our team and the data come from National Center for Biotechnology Information SNP database (https://www.ncbi.nlm.nih.gov/snp). In the current study, number of choosing SNPs in *CYP11B2* gene always rarely in 1 study, no polymorphisms covering the whole *CYP11B2* gene. Seven SNPs were identified for typing: SNP1 (rs28659182), SNP2 (rs10087214), SNP3 (rs73715282), SNP4 (rs542092383), SNP5 (rs4543), SNP6 (rs28491316), and SNP7 (rs7463212). These cover the entire *CYP11B2* gene for the first time (Fig. 1).

Blood samples were collected from all participants and anticoagulated with 2% ethylenediaminetetraacetic acid. Genomic DNA was extracted from peripheral blood leucocytes using the DNA extraction kit (ComWin Biotech Company, Beijing, China) and stored at –80°C. Genotyping was performed using the TaqMan SNP genotyping assay (Sequenom Company). Primers and probes were chosen from information available at the ABI website.

### 2.3 Statistical analysis

The Statistical Program for Social Science (SPSS version 17.0, Chicago, IL) was used to carry out statistical analysis. Differences in continuous data were compared by the *t* test. The Chi-square test was used to calculate categorical variables. Haplotypes data were analyzed using SHEsis online software (http://analysis.bio-x.cn/SHEsisMain.htm).^[17]^ Genotype distributions for each SNP in the control group were analyzed using the Chi-square test to see if they deviated from Hardy–Weinberg equilibrium (HWE). SNPs were excluded from further analysis if they showed evidence of deviation. Logistic regression models were used to analyze the independent effect of each genetic variant on the risk of EH. *P* values <.05 were considered to be statistically significant. In the haplotype-based case–control analysis, haplotypes with frequencies <0.01 were excluded.

## 3 Results

Table 1 summarizes the demographic characteristics of subjects. For all characteristics, significant differences were observed between normotensive controls and patients with EH. There was no deviation from HWE for the 7 SNPs in the normotensive group.

**Table 1:**
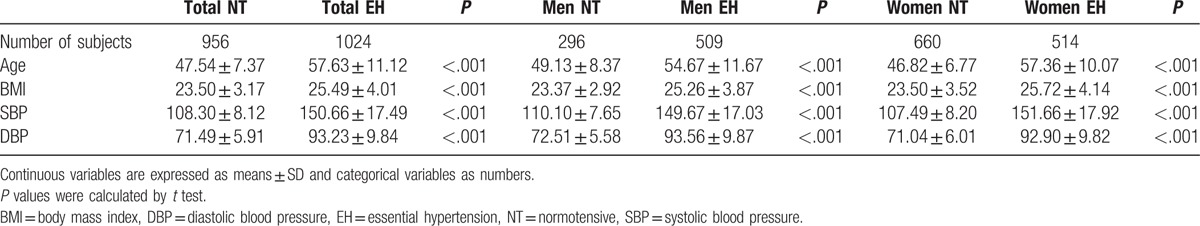
Demographic characteristics between normotensive and patients with essential hypertension.

Single-locus analyses of genotype and allele distributions in normotensive and EH patients for these SNPs are summarized in Table 2. *CYP11B2* SNP MAFs were 29.08%, 29.08%, 2.51%, 0.31%, 0.21%, 7.74%, and 32.43% in normotensive controls and 30.02%, 29.97%, 2.64%, 1.13%, 0.15%, 8.50%, and 33.48% in hypertensive patients for rs28659182, rs10087214, rs73715282, rs542092383, rs4543, rs28491316, and rs7463212, respectively. All genotype frequencies of these SNPs were in HWE (*P* > .05). The major allele G frequency of rs542092383 was found to be risk against hypertension [odds ratio (OR) 3.478, 95% confidence interval (95% CI) 1.407–8.597, *P* = .004]. The AG genotype frequency of rs542092383 was significantly associated with an increased risk of hypertension (OR 4.513, 95% CI 1.426–14.287, *P* = .010). The A allele frequency of rs542092383 was 0.9969 in normotensive controls and 0.9887 in patients with EH. The differences for other SNPs between these groups were not significant. The dominant (AA vs AG+GG) distribution of rs542092383 revealed a significant association (OR 6.815, 95% CI 2.275–20.416, *P* = .001) with EH risk.

**Table 2:**
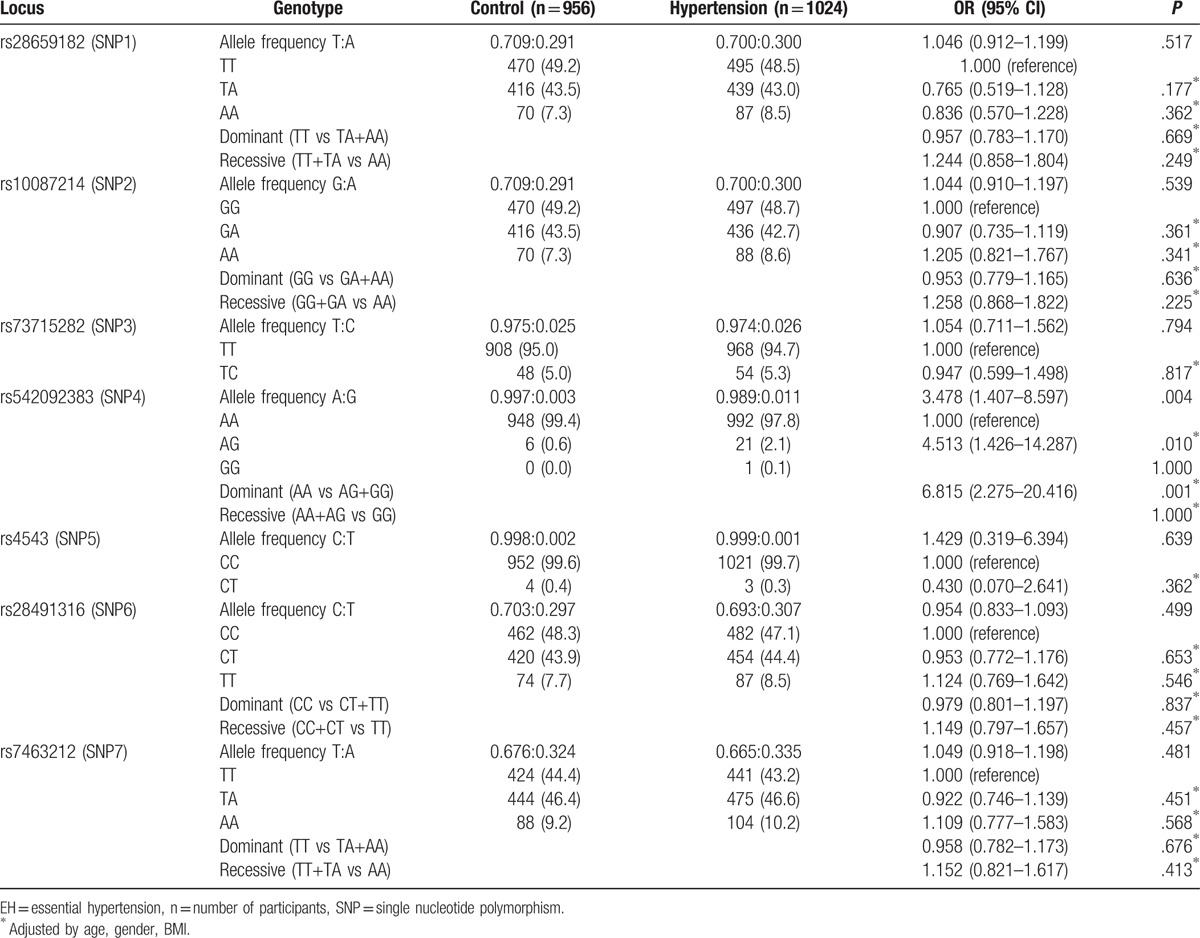
Genotypes of the 7 *CYP11B2* SNPs and the risk of EH.

The level of LD between the 7 SNPs was denoted by |D¢| and r2 values, which are presented in Table 3. In general, *r*^2^ is similar to D′ and it became more powerful even if one or both alleles are in low frequency.^[18]^ The findings indicate that all 7 SNPs are located in 1 haplotype block because all |D¢| values exceeded 0.9. *r*^2^ values for SNP1–SNP2, SNP1–SNP6, SNP1–SNP7, SNP2–SNP6, SNP2–SNP7, and SNP6–SNP7 were >0.8, indicating a strong linkage intensity. This also suggests that SNP1, SNP2, SNP6, and SNP7 could not be used to simultaneously construct the haplotype, and this analysis has previously been used in a similar study.^[19]^ Therefore, the haplotype was constructed using SNP7, which showed a larger MAF than SNP1, SNP2, or SNP6. The minor genotype frequency of SNP5 is too small, so we constructed a haplotype consisting of SNP3, SNP4, and SNP7. In the haplotype-based case–control analysis, the frequency of the T-G-T haplotype was significantly higher for EH patients than for controls (OR 5.729, 95% CI 1.889–17.371, *P* = .000495) in Table 4.

**Table 3:**
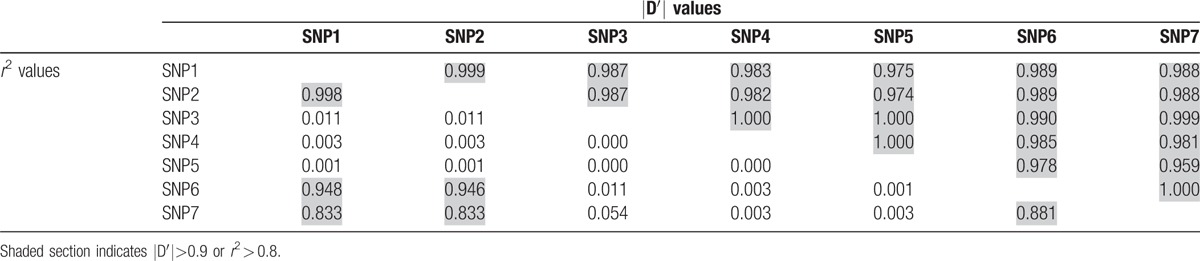
Pair linkage disequilibrium for 7 SNPs in the study subjects.

**Table 4:**
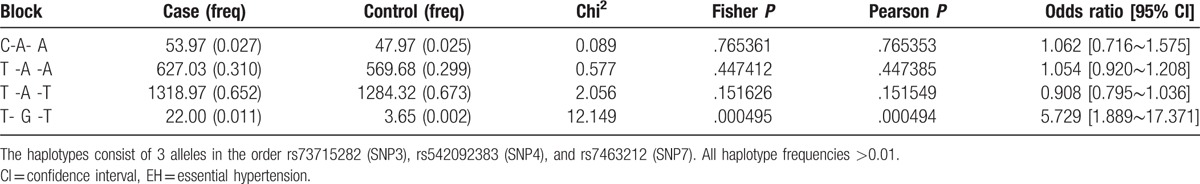
Haplotype frequencies of the 3 *CYP11B2* SNPs and the risk of EH.

## 4 Discussion

EH is a complex disease influenced by genetic and environmental factors and does not follow Mendelian genetic law. Risk factors include age, gender, family history of hypertension, being overweight, diabetes mellitus, excessive consumption of sodium, and smoking.^[20]^ Hypertension is responsible for most deaths caused by heart disease and stroke,^[21]^ suggesting that candidate genes for EH may also influence these other diseases.

Although genome-wide studies have provided valuable insights into the genetic basis of human complex diseases, SNPs discovered by GWAS account for only a small part of human complex diseases, and there is a question where is the “missing heritability.”^[22]^ Most of the heritability in complex diseases is not missing but has not previously been detected because the individual effects are too small to pass stringent significant tests, and causal variants have lower MAF than the SNPs explored.^[23]^ So, there is an advantage in the results of low-frequency variants over common variants.

In the candidate gene era, many SNPs within RAAS have been reported to be significantly associated with EH; however, the unbiased GWAS rarely identified that the SNPs within RAAS were associated with hypertension or BP traits, and the effect of RAAS polymorphisms may have been overestimated.^[24]^

Many studies have previously revealed that the RAAS has a key function in regulating human BP. Human cytochrome P450 family 11 subfamily B member 2 (CYP11B2) within RAAS is an essential enzyme in steroid hormone biosynthesis, catalyzing the last 3 reaction steps of aldosterone synthesis, and is an important player in the development of hypertension.^[25]^ Previously, studies suggested that several *CYP11B2* variations associate with hypertension, including C-344T, K173R, rs3802230, and rs10086846.^[26–28]^ The C-344T polymorphism of *CYP11B2* has been extensively studied in various populations, but the results of these studies are often inconsistent.^[13,29]^ Even there is no evidence to confirm that CYP11B2 (-344C/T) polymorphism within RAAS is associated with susceptibility of EH.^[30]^ Sequencing is the primary approach for the detection of low-frequency variants in complex diseases. It is conceivable that genetic and environmental factors, population variation, small sample sizes, patient and control selection, and limited genetic alleles contribute to the conflicting or even contradictory results.

Although GWAS has the advantage of analyzing the genetics of complex traits, the candidate gene approach is an important alternative strategy when the selected population is sufficiently large and relatively homogeneous.^[31]^

Our sample of 1024 EH patients and 956 normotensive controls gave sufficient capacity to use a candidate gene approach to obtain a stronger biological hint. Moreover, this study population represents an ethnically homogeneous community, which was entirely made up of northern Han Chinese living in Lanxi County, Heilongjiang Province. In addition, the results of HWE supported the selection of this study population.

This study is the first to report on the genetics of 7 *CYP11B2* polymorphisms in the north Chinese Han population. These SNPs are distributed from near the 3′ end of the gene (downstream) to near its 5′ end (upstream), and cover the entire gene. We found that the allelic frequencies of rs542092383 differed between controls and EH patients. The AG genotype of rs542092383 could therefore be used as a biomarker associated with an increased risk of hypertension. Moreover, the frequency of the T-G-T haplotype established by SNP3–SNP4–SNP7 was significantly lower for control subjects than for EH patients. These results come from G allele of rs542092383, which is a risk factor of EH, even if its frequency is low. Low-frequency variants showed that there is more power to detect risk variants than to detect protective variants in complex diseases. We got a larger OR value in dominant model than other genetic model.

LD is the nonrandom association of alleles at different loci and plays an important role in several aspects of human genetics.^[32]^ Understanding the structure and extent of LD is therefore very important in association mapping^[33]^ where the extent of the LD block is influenced by SNPs chosen by investigators. As summarized in Table 3, all 7 SNPs chosen in this study were located in the same haplotype block, and SNP1–SNP2–SNP6–SNP7 had a stronger linkage intensity than other SNPs; similar results were obtained for both male and female subjects.

There is increasing evidence that most recombination occurs in or around genes. Haplotype structure strongly affects recombination in maize,^[34]^ and LD spans larger segments in the rat and mouse than the human structure, which is characterized by much smaller blocks. Guryev et al^[35]^ found that recombination events were enriched within gene sequences, indicating that the probability of recombination in or around *CYP11B2* is greater than outside the gene. The map of the haplotype block can reflect the linkage intensity between some SNPs, but the actual recombination unit may possess a 3-dimensional structure. Synapsis occurs during prophase of meiosis I, which facilitates subsequent recombination events.^[36]^ It is inferred that differences in SNP linkage intensity may be caused by the synaptonemal complex, which is a proteinaceous structure formed by transverse filaments and lateral elements. During prophase of meiosis I, the connection between 3′ near gene region and 5′ near gene region may be short in space. The block of this investigation is merely a part of 1 larger human block, but self-similar phenomenon exists extensively in biology. The biological hints may be gained from results of this study.

## 5 Conclusion

We got results from the low-frequency variants that are more powerful to detect risk than to detect protective variants in EH. We used a candidate association approach to report an association between the AG genotype of *CYP11B2* rs542092383 with an increased risk of hypertension in the Han population of northern China. The T-G-T haplotype of *CYP11B2* was also associated with hypertension susceptibility. The intensity of linkage between the 3′ downstream region (SNP1-SNP2) to the 5′ upstream region (SNP6-SNP7) of *CYP11B2* was stronger than that at other locations of this gene.
